# Outcome Comparisons of Direct Coverage Versus Fenestration for an Isolated Left Vertebral Artery in Zone 2 TEVAR: A Retrospective Study

**DOI:** 10.31083/RCM44615

**Published:** 2026-03-10

**Authors:** Zuo Pu, Kun Fang, Jingbo Lu, Ying Zhang, Jiawei Zhao, Bowen Fan, Yi Liu, Mingyao Luo, Chang Shu

**Affiliations:** ^1^Peripheral Vascular Ward (Cardiac Surgery Ward 1), Fuwai Hospital Chinese Academy of Medical Sciences, 518001 Shenzhen, Guangdong, China; ^2^Department of Vascular Surgery, State Key Laboratory of Cardiovascular Disease, Fuwai Hospital, National Center for Cardiovascular Diseases, Chinese Academy of Medical Sciences and Peking Union Medical College, 100037 Beijing, China

**Keywords:** isolated left vertebral artery, physician-modified fenestration, aortic arch, thoracic endovascular aortic repair

## Abstract

**Background:**

Thoracic endovascular aortic repair (TEVAR) in Zone 2 frequently necessitates coverage of the isolated left vertebral artery (ILVA), a congenital vascular anomaly, to ensure adequate proximal sealing. However, the clinical requirement of ILVA revascularization remains uncertain. Thus, this study aimed to compare the outcomes between ILVA coverage and fenestration during Zone 2 TEVAR.

**Methods:**

We retrospectively analyzed the clinical records of patients with ILVA who underwent Zone 2 TEVAR between September 2010 and August 2023. Patients were divided into two groups: Coverage Group (n = 23) and Fenestration Group (n = 33). Baseline characteristics, surgical outcomes, and changes in left and right vertebral artery diameters pre- and postoperatively were compared. Continuous variables were compared using Student's *t*-test or Mann-Whitney U test, depending on the distribution. Categorical variables were analyzed using the chi-square test or Fisher's exact test.

**Results:**

The overall cohort had a mean age of 54.48 ± 10.31 years, with 89.29% of participants male and a mean body mass index (BMI) of 25.88 ± 3.5 kg/m^2^. The Fenestration Group was significantly older than the Coverage Group (56.82 ± 8.78 vs. 51.13 ± 11.56; *p* = 0.04). Technical success of the TEVAR was achieved in both groups in 98.21% of cases, with no perioperative mortality. Simultaneous left subclavian artery stenting was performed more frequently in the Fenestration Group (57.58% vs. 21.74%; *p* = 0.008). At discharge, patients in the Coverage Group demonstrated a significantly greater reduction in left vertebral artery diameter compared with the Fenestration Group (13.64% [5.52%, 22.4%] vs. 0 [–3.29%, 5.13%]; *p* < 0.001). The incidence of vertebral artery diameter reduction was significantly higher in the Coverage Group compared with the Fenestration Group (39.13% vs. 6.06%; *p* < 0.01). Follow-up computed tomography angiography demonstrated a greater reduction in left vertebral artery diameter in the Coverage Group (52.94% vs. 14.29%; *p* = 0.020), while occlusion rates were comparable between groups (29.41% vs. 4.76%; *p* = 0.070).

**Conclusions:**

Fenestration is associated with a lower incidence of postoperative ILVA diameter reduction compared with direct coverage during Zone 2 TEVAR. These findings highlight the potential benefit of ILVA revascularization and underscore the need for further validation in larger studies.

## 1. Introduction

Open surgical repair remains the gold standard for the treatment of aortic arch pathologies, particularly in patients with low-to-moderate surgical risk, where it is strongly recommended. However, for patients with high surgical risk, hybrid procedures or thoracic endovascular aortic repair (TEVAR) are considered reasonable alternatives and are classified as Class IIb recommendations [1]. With ongoing advancements in endovascular techniques and device design, TEVAR has been increasingly adopted in the treatment of aortic arch pathologies [2]. In TEVAR involving the aortic arch, effective proximal sealing frequently requires intentional coverage of one or more supra-aortic branches. In Zone 2 TEVAR, achieving an adequate proximal landing zone often necessitates coverage of the left subclavian artery (LSA). Additionally, anatomical variants, such as an isolated left vertebral artery (ILVA), may be encountered in patients undergoing TEVAR for aortic arch pathologies [3].

Historically, the ILVA was often covered during TEVAR without careful consideration of its contribution to cerebral circulation. However, recent studies have increasingly emphasized the importance of ILVA preservation, given its critical role in maintaining posterior circulation through the circle of Willis. Although several surgical strategies have been proposed to preserve the ILVA, the impact of physician-modified fenestration (PMF) on ILVA preservation and clinical outcomes remains insufficiently characterized [4]. Therefore, the objective of this study was to assess the clinical significance of ILVA preservation during Zone 2 TEVAR and its association with postoperative changes in vertebral artery diameter.

## 2. Materials and Methods

### 2.1 Study Population

This retrospective, single-center study included patients with aortic arch pathologies who underwent TEVAR at our institution between September 2010 and August 2023. Inclusion criteria were: (1) diagnosis of aortic arch pathology, including but not limited to acute aortic syndrome (AAS), aortic aneurysm, or related conditions; (2) planned Zone 2 TEVAR with coverage of the LSA, potentially involving coverage of the ILVA; and (3) preoperative computed tomography angiography (CTA) confirming the presence of an ILVA. Exclusion criteria were: (1) poor-quality CTA imaging that could not be reliably evaluated, and (2) patients deemed unsuitable for TEVAR due to other medical reasons. AAS refers to a spectrum of life-threatening conditions, including aortic dissection, intramural hematoma, and penetrating aortic ulcers, which are recognized indications for TEVAR under current clinical guidelines [2, 5]. This study was approved by the Ethics Committee of Fuwai Hospital. Owing to the retrospective design, the requirement for informed consent was waived.

Two experienced radiologists independently measured vascular diameters using Endosize software (Therenva, Rennes, France). To minimize measurement errors, diameters were assessed 2 cm from the origin of both vertebral arteries. Postoperative occlusion of the isolated left vertebral artery was defined as the absence of intraluminal blood flow following TEVAR. Postoperative stenosis of the isolated left vertebral artery was defined as a ≥30% reduction in diameter compared with preoperative measurements. Primary technical success was defined as successful deployment of the stent graft, excluding aortic pathology, with no conversion to open surgery and no surgery-related mortality. In the Fenestration Group, preservation of ILVA patency was additionally required to qualify as a technical success, reflecting the goal of revascularization.

### 2.2 Procedural Techniques

**Fig. 1.**
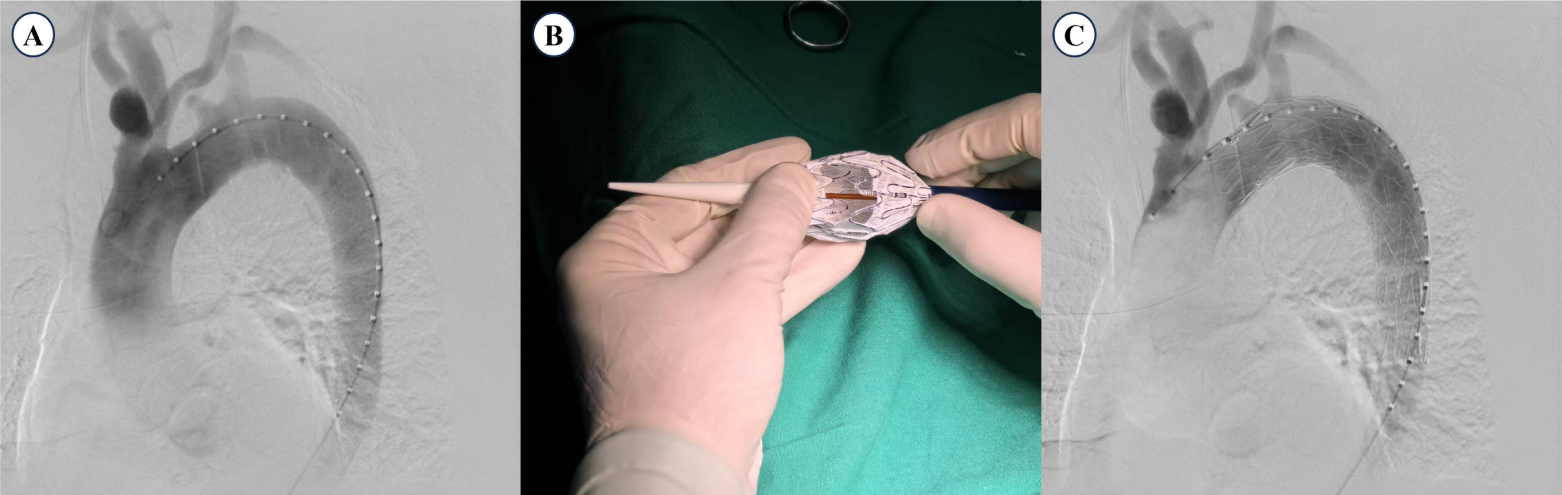
**Procedural steps for the PMF technique in TEVAR**. (A) Preoperative DSA showing aortic pathology and vertebral artery anatomy. (B) Fenestration of the ILVA and LSA openings performed with electrocautery, guided by the “∞” radiopaque marker for accurate positioning. (C) Deployment of the stent graft with subsequent confirmation of vessel patency on DSA. PMF, physician-modified fenestration; DSA, digital subtraction angiography; TEVAR, thoracic endovascular aortic repair; ILVA, isolated left vertebral artery; LSA, left subclavian artery.

All procedures were performed under general anesthesia. Standard disinfection and draping were applied to the bilateral femoral and left brachial artery regions. A 5F gold marker pigtail catheter was introduced through the femoral artery into the ascending aorta for digital subtraction angiography (DSA) (Fig. 1A). Measurements of the aortic lesion and its morphological characteristics were obtained from CTA and DSA images. The aortic stent graft was selected with a diameter oversized by 15–20% relative to the measured aortic diameter. Systemic heparinization was administered in all patients. An Ankura thoracic aortic-covered stent (Lifetech Scientific Co., Ltd., Shenzhen, Guangdong, China) was used in every procedure. This device features a longitudinally distributed metallic support structure that provides additional reinforcement along the greater curvature of the aorta and incorporates a radiopaque marker to delineate the transition between the bare metal and covered segments. When aligned perpendicularly to the X-ray beam, the marker appears as a “∞” symbol; and when parallel, it appears as a “—” symbol.

**Fig. 2.**
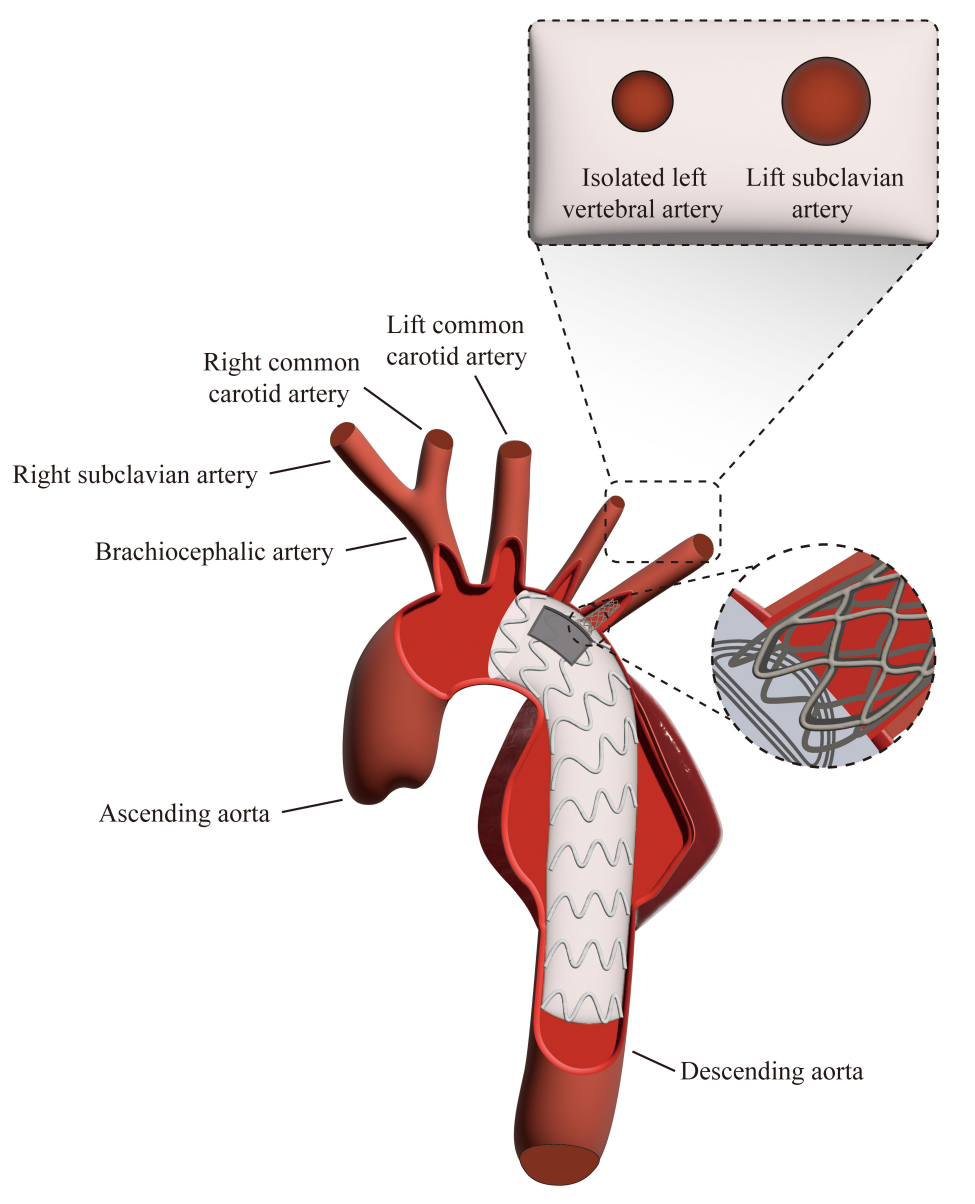
**Illustration of TEVAR procedure with PMF and bridging stent**. The diagram demonstrates the key steps of TEVAR with PMF for ILVA preservation and bridging stent placement for the LSA.

Upon confirmation of the “∞” radiopaque marker, the outer sheath of the aortic stent was retracted 4–5 cm to expose its proximal end, and fenestration was performed using an electrocautery pen (Fig. 1B). The fenestration covered the openings of the left subclavian artery and isolated the left vertebral artery. The sheath was then advanced using the bunding technique until it was repositioned in its original location. The stent graft was subsequently delivered to the aortic lesion over a Lunderquist super-stiff guidewire (William Cook Europe, Bjaeverskov, Denmark). Deployment was performed with the covered segment positioned adjacent to the posterior edge of the left common carotid artery (Fig. 1C). A pigtail angiographic catheter was reintroduced to perform DSA and confirm patency of the supr-aortic branches. In the Coverage Group, the procedural steps were similar except that no fenestration or bridging stents were performed. Both the ILVA and LSA were intentionally covered during Zone 2 TEVAR without revascularization. Endoleaks detected intraoperatively or during follow-up were classified as follows: Type Ia, proximal endoleak caused by inadequate sealing at the landing zone; Type II, retrograde flow from branch arteries arising from the excluded segment, most commonly via the left subclavian artery; and Type III, leakage due to stent graft failure, such as component separation or fabric disruption [6]. To preserve LSA patency, we generally consider implanting a bridging stent (Fig. 2). However, in cases of marked tortuosity of the proximal subclavian artery, dissection involving the subclavian artery, or aneurysmal disease at its origin, bridging stent implantation was deemed unsuitable.

All patients with a history of hyperlipidemia or diabetes mellitus received standard lipid-lowering and antidiabetic treatment during the perioperative period, applied consistently across both groups. In the Fenestration Group, antithrombotic therapy was tailored according to the diameter of the bridging stent: patients with stents ≤8 mm received dual antiplatelet therapy (DAPT) for 3 months postoperatively, while those with stents >8 mm received single antiplatelet therapy. Routine postoperative antiplatelet therapy was not administered in the Coverage Group or in the patients without stent implantation.

### 2.3 Statistical Analysis

Continuous variables were expressed as mean ± standard deviation for normally distributed data or as median (Q1, Q3) for non-normally distributed data, and were compared between groups using Student’s *t*-test or the Mann-Whitney U test, as appropriate. Categorical variables were presented as frequencies and percentages, and group comparisons were performed using Fisher’s exact test or the chi-square test. All *p*-values were two-sided, with statistical significance defined as *p* < 0.05. Statistical analyses were conducted using SPSS version 26.0 (IBM Corp., Armonk, NY, USA).

### 2.4 Follow-Up

Patients were followed up through outpatient visits and telephone interviews. The follow-up duration ranged from 1 month to 13 years. All patients were recommended to undergo CTA prior to discharge and annually thereafter, or earlier if clinically indicated, to monitor for complications such as endoleaks, stent migration, or changes in vertebral artery patency.

## 3. Results

### 3.1 Baseline Characteristics

**Fig. 3.**
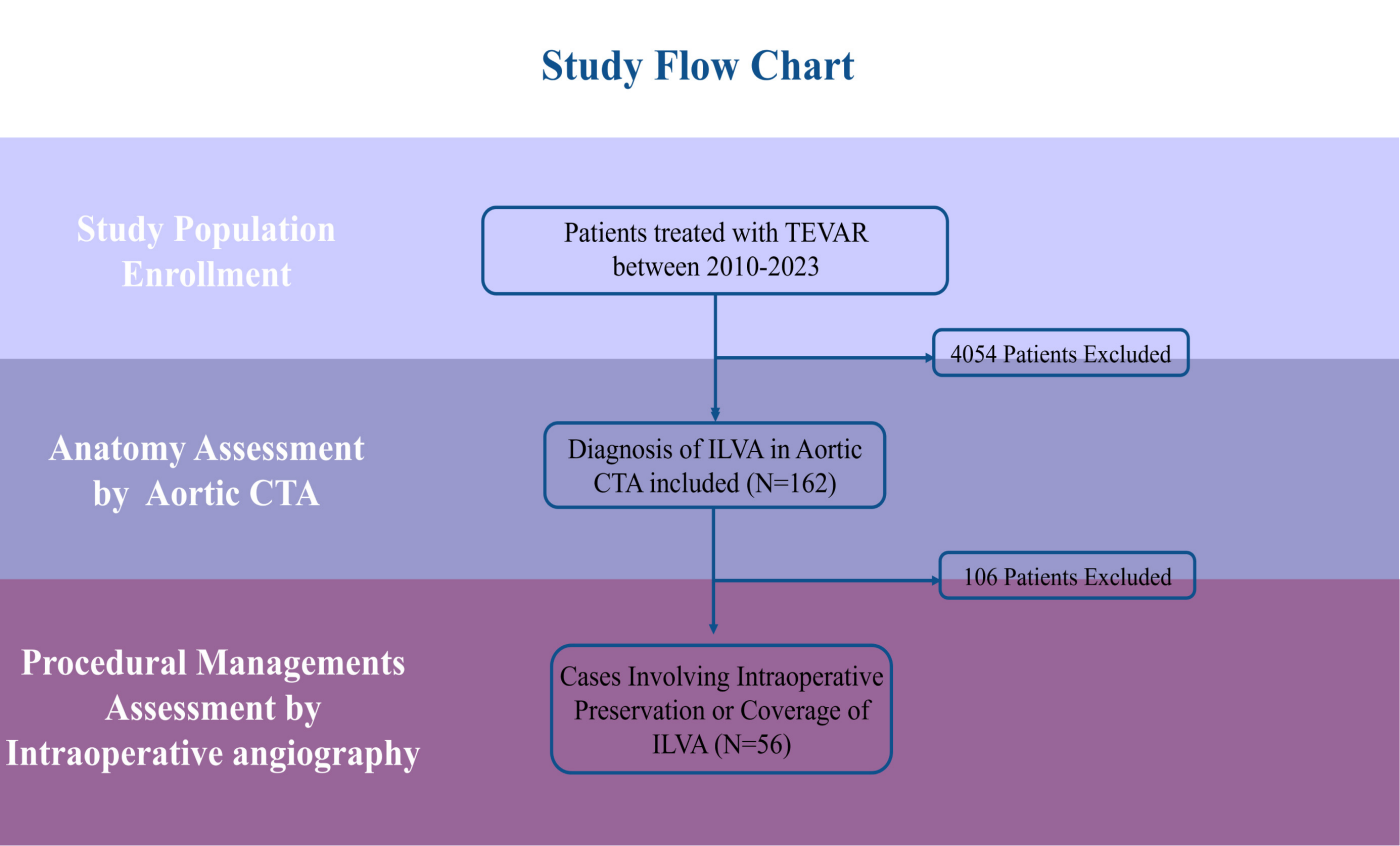
**Patient selection and study flowchart**. CTA, computed tomography angiography.

A total of 56 patients with aortic arch disease and ILVA who underwent TEVAR were included in this study (Fig. 3). Patients were divided into either the Coverage Group (N = 23) or the Fenestration Group (N = 33), depending on whether fenestration was performed (Table 1). The mean age of the overall cohort was 54.48 ± 10.31 years, with 89.29% male, and the mean body mass index was 25.88 ± 3.5 kg/m^2^. The primary diagnoses were acute aortic syndrome (55.36%) and aortic ulcer (21.43%), and chest pain was the most frequent presenting symptom (64.29%). Patients in the Fenestration Group were significantly older than those in the Coverage Group (56.82 ± 8.78 vs. 51.13 ± 11.56, *p* = 0.04). No significant differences were observed between the two groups with respect to gender, presenting symptoms, medical history, or underlying diagnosis. Preoperative CTA showed comparable left vertebral artery (LVA) diameters between the Coverage and Fenestration Groups (3.07 ± 0.69 mm vs. 3.06 ± 0.64 mm, *p* = 0.97), as well as comparable right vertebral artery (RVA) diameters (3.85 ± 0.69 mm vs. 3.66 ± 0.40 mm, *p* = 0.25).

**Table 1.** **Baseline characteristics**.

Parameters		Total (N = 56)	Coverage Group (N = 23)	Fenestration Group (N = 33)	*p*-value
Age (years)		54.48 ± 10.31	51.13 ± 11.56	56.82 ± 8.78	0.04
Male, n (%)		50 (89.29)	23 (100)	27 (81.82)	0.09
Body Mass Index (kg/m^2^)		25.88 ± 3.50	25.47 ± 3.86	26.17 ± 3.25	0.47
Clinical Presentation, n (%)					0.20
	Asymptomatic	15 (26.79)	5 (21.74)	10 (30.30)	
	Chest Pain	36 (64.29)	14 (60.87)	22 (66.67)	
	Abdominal Pain	5 (8.93)	4 (17.39)	1 (3.03)	
Medical History, n (%)					
	Hyperlipidemia	18 (32.14)	6 (26.09)	12 (36.36)	0.42
	Hypertension	44 (78.57)	19 (82.61)	25 (75.76)	0.78
	Diabetes	5 (8.93)	0	5 (15.15)	0.14
	Coronary Artery Disease	8 (14.29)	2 (8.70)	6 (18.18)	0.54
	History of Stroke	2 (3.57)	2 (8.70)	0	0.16
	Chronic Obstructive Pulmonary Disease	1 (1.79)	0	1 (3.03)	1.00
	Smoking	26 (46.43)	8 (34.78)	18 (54.55)	0.14
	History of Open Surgery	1 (1.79)	1 (4.35)	0	0.41
Left Ventricular Ejection Fraction (%)		61.61 ± 4.93	61.61 ± 5.84	61.61 ± 4.29	1.00
Clinical Diagnosis, n (%)					0.12
	Acute Aortic Syndrome	31 (55.36)	13 (56.52)	18 (54.55)	
	Aortic Ulcer	12 (21.43)	2 (8.70)	10 (30.30)	
	True Thoracic Aortic Aneurysm	5 (8.93)	4 (17.39)	1 (3.03)	
	Pseudo Thoracic Aortic Aneurysm	3 (5.36)	1 (4.35)	2 (6.06)	
	Chronic Type B Aortic Dissection	5 (8.93)	3 (13.04)	2 (6.06)	
Preoperative Aortic CTA					
	Left Vertebral Artery Diameter (mm)	3.06 ± 0.66	3.07 ± 0.69	3.06 ± 0.64	0.97
	Right Vertebral Artery Diameter (mm)	3.74 ± 0.54	3.85 ± 0.69	3.66 ± 0.40	0.25

### 3.2 Procedural Outcomes

The procedural outcomes are detailed in Table 2. The overall technical success rate of TEVAR was 98.21%, and no perioperative mortality occurred. A significantly higher proportion of patients in the Fenestration Group underwent simultaneous left subclavian artery stenting compared with the Coverage Group (57.58% vs. 21.74%, *p* = 0.008). No significant differences were observed between the two groups with respect to endoleak rates, perioperative complications, or postoperative length of stay.

**Table 2.** **Procedural outcomes**.

Parameters	Total (N = 56)	Coverage Group (N = 23)	Fenestration Group (N = 33)	*p*-value
Technical success rate, n (%)	55 (98.21)	23 (100)	32 (96.97)	1.000
Simultaneous LSA stenting, n (%)	24 (42.86)	5 (21.74)	19 (57.58)	0.008
Perioperative mortality, n (%)	0	0	0	1.000
Endoleak, n (%)	4 (7.14)	2 (8.70)	2 (6.06)	1.000
Postoperative length of stay, days	5.29 ± 1.92	5.39 ± 2.06	5.21 ± 1.85	0.730
Pre-discharge CTA measurements, n (%)	54 (96.43)	23 (100.00)	31 (93.94)	0.510
LVA diameter, mm	2.79 ± 0.81	2.47 ± 1.01	3.02 ± 0.52	0.020
LVA reduction in diameter, mm	0.10 (0, 0.5)	0.3 (0.15, 0.70)	0 (–0.10, 0.15)	<0.001
Change in LVA diameter (% decrease)	3.03% (0, 13.61)	13.64% (5.52, 22.4)	0 (–3.29, 5.13)	<0.001
RVA diameter, mm	3.75 ± 0.56	3.98 ± 0.59	3.58 ± 0.48	0.009
RVA reduction in diameter, mm	0 (–0.37, 0.20)	–0.10 (–0.40, 0.05)	0.10 (–0.25, 0.35)	0.030
Change in RVA diameter (% decrease)	0 (–10.26, 5.71)	–2.56% (–10.98, 0.98)	2.38% (–6.98, 9.41)	0.050
LVA occlusion rate, n (%)	2 (3.57)	2 (8.70)	0	0.160
Incidence of vertebral artery diameter reduction, n (%)	11 (19.64)	9 (39.13)	2 (6.06)	<0.01

LSA, left subclavian artery; LVA, left vertebral artery; RVA, right vertebral artery.

A total of 54 patients (96.43%) underwent CTA at discharge. Compared with preoperative and postoperative measurements, the Coverage Group exhibited a significantly greater reduction in LVA diameter at discharge than that of the Fenestration Group 13.64% [5.52, 22.4%] vs. 0 [–3.29%, 5.13%], *p* < 0.001). The RVA diameter increased by 2.56% in the Coverage Group, whereas the Fenestration Group demonstrated a 2.38% decrease. Although LVA occlusion rates at discharge were similar between groups (8.7% vs. 0%, *p* = 0.16), the incidence of LVA diameter reduction was significantly higher in the Coverage Group than in the Fenestration Group (39.13% vs. 6.06%, *p* < 0.01).

### 3.3 Follow-up Outcomes

Among the 38 patients who underwent follow-up CTA, the median follow-up CTA interval was 41.38 (21.42, 73.33) months. The follow-up CTA interval was significantly longer in the Coverage Group than in the Fenestration Group (79.33 [34.25, 106.73] vs. 36.43 [19.67, 55.67] months, *p* = 0.010). The incidence of complications during follow-up was comparable between groups (17.39% vs. 9.09%, *p* = 0.61). In the Fenestration Group, one patient experienced an ischemic stroke and two patients developed endoleaks on follow-up CTA. In the Coverage Group, adverse events included one sudden unexplained death, one type II endoleak from the LSA that was treated with coil embolization at 9 months, one reintervention with repeat TEVAR for a new distal aortic ulcer, and one case of LSA stent occlusion. Additionally, a type III endoleak occurred in one Coverage Group patient, which resolved spontaneously at the 3-month CTA follow-up. All endoleaks were either self-limiting or successfully treated, and no long-term adverse events were reported. No patient in the Coverage Group required carotid-subclavian bypass surgery for posterior circulation hypoperfusion or upper limb ischemia.

**Fig. 4.**
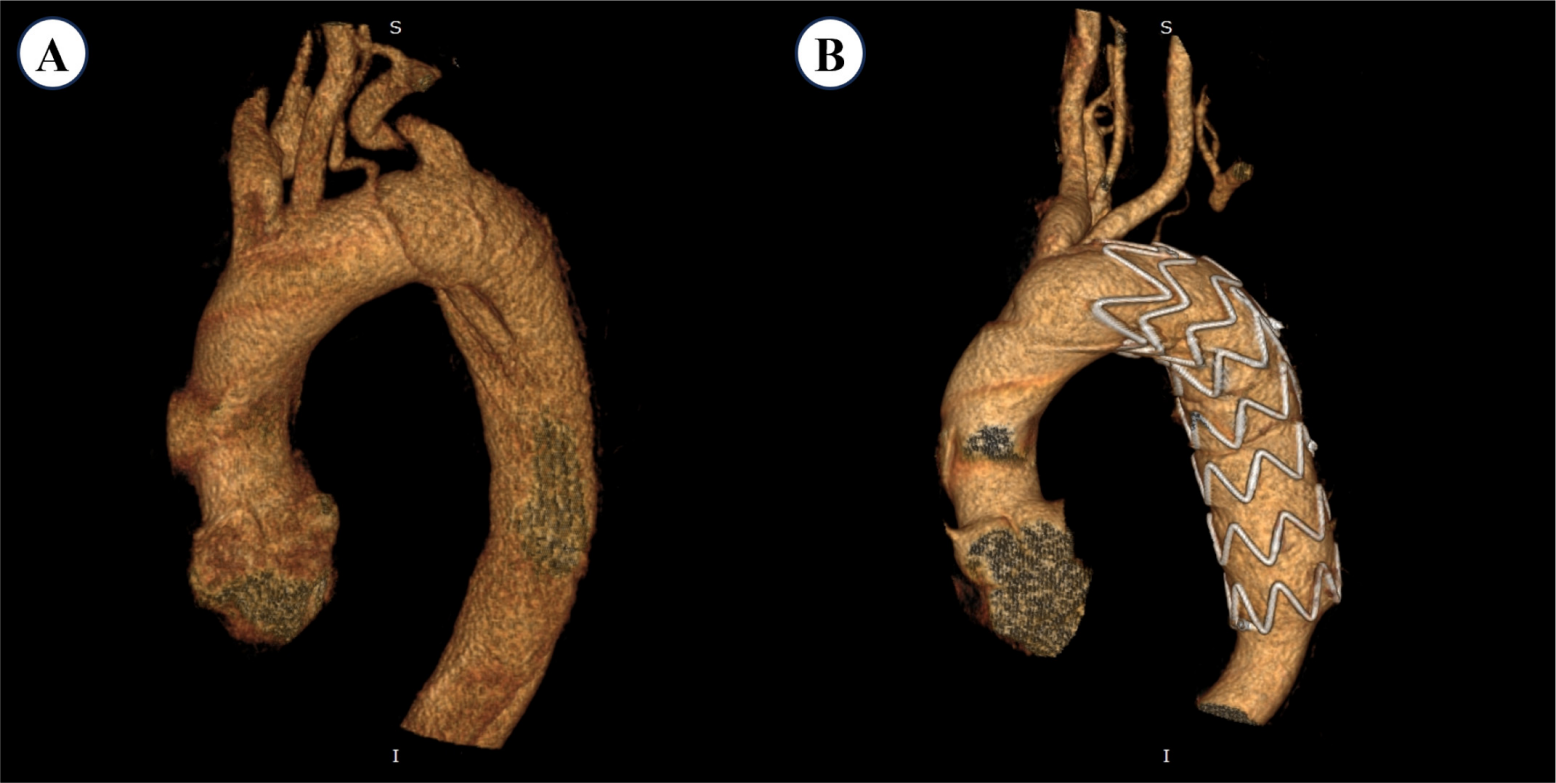
**Preoperative and postoperative 3D-CTA images of a patient in the Coverage Group**. (A) Preoperative image showing a prominent ILVA arising directly from the aortic arch, with normal caliber and course. (B) Four-year postoperative image showing a marked reduction in ILVA diameter, indicating chronic hypoperfusion, and absence of contrast opacification in the LSA. S, superior; I, inferior.

A total of 38 patients underwent follow-up CTA, including 17 in the Coverage Group and 21 in the Fenestration Group, with a mean interval of 23.29 ± 32.09 months , see Table 3. At the final CTA, the LVA diameter was significantly greater in the Fenestration Group than in the Coverage Group (2.93 ± 0.79 mm vs. 1.78 ± 1.40 mm, *p* = 0.006), while the RVA diameter was larger in the Coverage Group (4.20 ± 0.60 mm vs. 3.62 ± 0.53 mm, *p* = 0.003). Compared with the preoperative values, the degree of LVA diameter reduction at follow-up was significantly greater in the Coverage Group (28.57% [10%, 100%]) than in the Fenestration Group (0 [–3.03%, 8.33%], *p* = 0.005). The incidence of LVA diameter reduction was also higher in the Coverage Group than in the Fenetration Group (52.94% vs. 14.29%, *p* = 0.020). However, LVA occlusion rates did not differ significantly between groups (29.41% vs. 4.76%, *p* = 0.070). A representative case illustrating ILVA diameter reduction and absence of contrast opacification of the LSA at four-year follow-up is shown in Fig. 4.

**Table 3.** **Follow-up outcomes**.

Parameters	Total (N = 38)	Coverage (N = 17)	Fenestration (N = 21)	*p*-value
Follow-up CTA interval, months	41.38 (21.42, 73.33)	79.33 (34.25, 106.73)	36.43 (19.67, 55.67)	0.010
LVA diameter at follow-up, mm	2.42 ± 1.23	1.78 ± 1.40	2.93 ± 0.79	0.006
LVA reduction, mm	0.25 (–0.10, 1)	0.80 (0.30, 2.3)	0 (–0.10, 0.3)	0.007
LVA decrease, %	8.22 (–2.58, 28.57)	28.57 (10, 100)	0 (–3.03, 8.33)	0.005
RVA diameter at follow-up, mm	3.88 ± 0.63	4.20 ± 0.60	3.62 ± 0.53	0.003
RVA reduction, mm	–0.20 (–0.57, 0.17)	–0.30 (–0.90, –0.2)	0 (–0.20, 0.4)	0.005
RVA decrease, %	–4.89 (–16.31, 3.75)	–10.34 (–24.39, –5.13)	0 (–5.41, 11.43)	0.003
Incidence of LVA diameter occlusion, n (%)	6 (15.79)	5 (29.41)	1 (4.76)	0.070
Incidence of LVA diameter reduction, n (%)	12 (31.58)	9 (52.94)	3 (14.29)	0.020

## 4. Discussion

Following the carotid arteries, the vertebral arteries play a critical role in cerebral perfusion and constitute a crucial component of the circle of Willis. Posterior circulation infarctions account for approximately 25–30% of all ischemic strokes [5]. Prior studies have shown that reduced blood flow in the left vertebral and subclavian arteries increases the risk of symptomatic vertebrobasilar insufficiency, spinal cord injury (SCI), and stroke [7, 8, 9]. In the management of complex thoracic aortic pathologies, particularly in patients with an ILVA, achieving an adequate proximal landing zone often requires coverage of both the LSA and LVA. The isolated left vertebral artery typically arises directly from the aortic arch between the left common carotid artery and LSA. This anatomical variant represents the second most common variation of the supra-aortic trunks, with a reported prevalence of approximately 4.81% [10]. Current guidelines recommend that in patients with an ILVA arising directly from the thoracic aorta, vertebral artery revascularization should be considered when TEVAR involves its origin [1]. Compared with LSA revascularization, revascularization of an isolated left vertebral artery presents greater technical challenges during TEVAR.

In recent years, recognition of the importance of preserving the ILVA during TEVAR has grown. Several studies have investigated various strategies to achieve this objective in the setting of complex thoracic aortic disease. Yang *et al*. [11] demonstrated that hybrid procedures combining TEVAR, ILVA transposition, and left common carotid-subclavian artery bypass are both safe and feasible for managing thoracic aortic pathologies involving the ILVA. Luo *et al*. [12] reported the use of a physician-modified Castor branched stent with fenestration to reconstruct the ILVA during aortic arch surgery. Their findings indicated that the PMF technique with the Castor stent, performed under local anesthesia, was both safe and effective [12]. Shen *et al*. [13] highlighted the feasibility of *in situ* fenestration for ILVA reconstruction in complex aortic arch disease. This method was also shown to be both safe and effective, although it required direct ILVA exposure and sheath placement via puncture [13]. In a multicenter retrospective study, Zhang *et al*. [4] compared three techniques: a novel chimney approach using right brachial-left brachial crossover, external fenestration, and arterial transposition. At our center, we utilized the PMF technique to preserve the ILVA in cases of complex thoracic aortic disease. In the present study, fenestration did not significantly affect overall surgical outcomes, and no substantial differences were observed in endoleak incidence between groups.

Most previous studies have focused on the technical methods and immediate outcomes of ILVA preservation, whereas limited attention has been directed toward the hemodynamic consequences of ILVA preservation after TEVAR. In this study, we innovatively evaluated the hemodynamic impact of preserving the ILVA using PMF during TEVAR. Our findings showed that, for patients undergoing Zone 2 TEVAR involving the ILVA, the Fenestration Group had a significantly lower incidence of postoperative ILVA stenosis compared with the Coverage Group. Previous research has demonstrated that preoperative revascularization of the LSA in patients requiring LSA coverage during TEVAR reduces the risk of stroke and SCI [14, 15]. This protective effect was likely due to the maintenance of posterior circulatory perfusion through the left vertebral artery. Therefore, preserving the ILVA during TEVAR is particularly important for patients with dominant left vertebral arteries or incomplete circles of Willis [7, 16]. Our study also found that in the Coverage Group, the non-stenosis rate was 52.17%. We hypothesize that although the left vertebral artery origin was covered, the relatively short coverage length and incomplete apposition of the stent graft to the aortic wall may have minimized the hemodynamic impact on the left vertebral artery in these patients.

Owing to varying levels of understanding of the disease, not all interventional vascular specialists at our center routinely use fenestration techniques to preserve ILVA flow. However, as on-table fenestration techniques have advanced in recent years and in the absence of dedicated devices for managing aortic arch pathologies with vascular anomalies, we recommend PMF as an effective treatment option. Based on our experience and the anatomical characteristics of the aortic arch branches, the origin of the ILVA is typically located along the centerline of the aortic arch, within the contour of the LSA opening on the greater curvature of the arch. Therefore, if adequate blood flow to the LSA is maintained, coverage of the ILVA origin is unlikely.

### Limitations

This study has several limitations. First, being a retrospective, single-center study, inherent biases related to patient selection, data collection, and analysis may affect the generalizability of our findings. For instance, patients in the Fenestration Group were significantly older, which may reflect a clinical preference for preserving posterior circulation in older patients who have more comorbidities, thus introducing potential selection bias. Second, the relatively small sample size may have limited the statistical power to detect subtle differences between the coverage and fenestration groups. Third, although our study focused on the hemodynamic impact of ILVA preservation, neurological events were not predefined as primary endpoints, and no postoperative neuroimaging, such as brain MRI, was routinely performed. Finally, long-term functional outcomes, including cerebrovascular events and patient-reported quality of life, were not evaluated. These additional endpoints may provide further insights into the clinical significance of ILVA revascularization.

## 5. Conclusions

In patients with ILVA undergoing Zone 2 TEVAR, fenestration was associated with a lower incidence of postoperative ILVA diameter reduction compared to direct coverage. These findings suggest a potential benefit of ILVA revascularization; however, due to the study’s limited sample size, further research is needed to confirm these results.

## Availability of Data and Materials

All data generated or analyzed during this study are included in this published article.
